# Study protocol for Valor Barroso: a mixed-method approach to dietary–physical activity patterns in Barroso, Portugal

**DOI:** 10.1186/s13690-026-01900-8

**Published:** 2026-03-26

**Authors:** Anna Carolina Cortez-Ribeiro, José Carlos Ribeiro, Maria José Alves, Vera Ferro-Lebres

**Affiliations:** 1 CIMO, LA SusTEC, Instituto Politécnico de Bragança, Campus de Santa Apolónia, Bragança, 5300- 253 Portugal; 2 Centro de Investigação em Actividade Física Saúde e Lazer (CIAFEL), Faculdade de Desporto, Universidade do Porto, Rua Dr. Plácido da Costa, 91, Porto, 4200-450 Portugal; 3 Laboratório para a Investigação Integrativa e Translacional em Saúde Populacional (ITR), Universidade do Porto, Rua das Taipas, 135, Porto, 4050-600 Portugal; 4 Centro de Valorização e Transferência de Tecnologia da Água, AquaValor, Rua Dr. Júlio Martins, 1, Chaves, 5400-342 Portugal; 5 LiveWell - Research Centre for Active Living & Wellbeing, Instituto Politécnico de Bragança, Campus de Santa Apolónia, Bragança, 5300- 253 Portugal

**Keywords:** Dietary patterns, Physical activity, Lifestyle, Rural population, Public health

## Abstract

**Aim:**

This community-based mixed-methods study aims to evaluate the dietary habits and physical activity patterns of the Barroso population in northern Portugal.

**Methods:**

This study was divided into two phases. The quantitative phase will be a cross-sectional study involving 416 participants from different age groups, including children, adolescents, adults, and elderly individuals. Sociodemographic and clinical, including anthropometric measurements, blood pressure, and quality of life, will be collected. Physical activity will be assessed wGT3X+ Actigraph accelerometers and validated questionnaires, whereas dietary habits will be evaluated via food frequency questionnaires, 24-hour dietary recalls, and food diaries. In the qualitative phase, participants will be selected through stratified sampling and ethnographic analysis will be conducted through observations and semi-structured interviews to explore the cultural, social, and environmental factors influencing dietary habits and physical activity in the local population.

**Discussion:**

By integrating objective measures of physical activity and diet, such as accelerometers and validated questionnaires, with qualitative data on cultural and traditional practices, this study aims to provide a more comprehensive understanding of lifestyle behaviors in the region and their associations with health. Additionally, the study investigated how geographical, socioeconomic, and cultural contexts influence these dietary and physical activity behaviors. The conclusions drawn will help develop public health interventions to improve health outcomes in rural communities and will contribute to the development of more effective strategies for similar populations.

**Table:** 

Text box 1. Contributions to the literature
• There is limited evidence on the assessment of dietary and physical activity habits in rural Portuguese populations, which are often underrepresented in public health research.
• This protocol provides a detailed description of a mixed-methods study (quantitative and qualitative) to evaluate diet and physical activity in rural communities.
• The collection and integration of quantitative and qualitative data will enable a deeper understanding and a robust picture of lifestyle behaviors in the Barroso region.
• The findings will support public health interventions and policies tailored to the population, respecting cultural traditions while promoting healthier lifestyles in rural communities.

## Background

Dietary patterns and physical activity levels are widely recognized as key determinants of public health and directly influence the risk of developing noncommunicable chronic diseases (NCDs), such as obesity, type 2 diabetes mellitus, hypertension, and cardiovascular diseases [1, 2]. These conditions are among the leading causes of morbidity and mortality worldwide, accounting for approximately five million deaths annually, many of which could be prevented through the adoption of healthier lifestyles [3].

Despite the widely disseminated guidelines from the World Health Organization (WHO) recommending regular physical activity, 27.5% of adults globally, and 27% of the Portuguese population do not meet the minimum recommended levels [4, 5]. This situation is further exacerbated by the increasing prevalence of sedentary behavior, which has been associated with an increased risk of metabolic syndrome and other NCDs [6].

Diet is also a crucial modifiable factor in the prevention of these diseases. It is estimated that inadequate diets are responsible for approximately 11 million deaths annually and over 255 million disability-adjusted life years (DALYs) [1, 7]. Traditional dietary systems, such as the Mediterranean diet, offer well-documented health benefits, emphasizing the consumption of fresh, local, and minimally processed foods [8, 9]. However, food globalization and the industrialization of production systems have led to a homogenization of eating habits, often replacing traditional products with ultra-processed foods that are typically low in nutritional value [10–12].

This nutritional transition is particularly evident in urban areas, and is accompanied by lifestyle changes related to urbanization, shifts in labor patterns, transportation modes, increased technology use, and evolving cultural values [13–15]. In contrast, many rural residents maintain traditional practices [12, 16], both in terms of diet and physical activity, shaped by structural, geographical, cultural, and socioeconomic factors. It is estimated that 43% of the global population resides in rural areas, including approximately 1.2 million people in Portugal [17, 18]. However, most existing research focuses on urban settings, limiting a deeper understanding of rural Portuguese realities, especially in the Barroso region.

Located in northern Portugal and comprising the municipalities of Montalegre and Boticas, the Barroso region is characterized by unique sociocultural and environmental features. In 2018, it was designated as a Globally Important Agricultural Heritage System (GIAHS) by the Food and Agriculture Organization (FAO) of the United Nations, in recognition of its preservation of traditional and sustainable agricultural and pastoral practices, which were largely maintained due to the region’s historical and geographical isolation [19]. Strong community cohesion, resource sharing, and the continuity of traditional habits continue to shape daily life and sustain a deeply rooted cultural identity.

In this context, the Valor Barroso study aims to comprehensively characterize dietary and physical activity patterns among the local population, using a mixed-methods approach. The goal is to determine the impact of these lifestyle factors on health outcomes and to inform future public health interventions. The specific objectives include the following:

- Identify the levels of physical activity and sedentary behavior among different population groups in Barroso;
- Investigate the influence of agricultural labor and geographic constraints on daily physical activity levels;
- Compare regional health indicators with national data to determine the prevalence of NCDs linked to lifestyle factors;
- Analyze the dietary habits and food consumption patterns of the local population;
- Conduct an ethnographic exploration of traditional lifestyle, focusing on cultural, social, and environmental influences on dietary intake and physical activity.

## Methods

### Study design

The present study is a community-based mixed-method study that will evaluate the dietary and physical activity patterns of the Barroso population. Phase 1 consists of quantitative analysis, using accelerometers, validated questionnaires, and anthropometric measurements. Phase 2 focuses on qualitative ethnographic through semi-structured interviews and participant observations.

A mixed methods approach is ideal for this study because it combines the strengths of both methods: qualitative data reveal traditional patterns and explore the cultural, social and environmental factors influencing dietary practices and physical activity, experiences directly from the population; and the quantitative phase allows the generalization of findings to a larger population.

We will use a triangulation design for concurrent collection and analysis. This allows us to compare findings from both phases and identify contradictions, gaps or biases in the data, enriching the overall understanding of the study outcomes.

The study is a collaborative effort involving, within the Polytechnic Institute of Bragança (IPB), the Mountain Research Centre (CIMO), the Associated Laboratory for Sustainability and Technology in Mountain Regions (SUSTEC), and AquaValor – Centre for the Valorization and Transfer of Water Technology; and, within the University of Porto, the Research Centre in Physical Activity, Health and Leisure (CIAFEL) and the Laboratory for Integrative and Translational Research in Population Health (ITR). The project is supported by the Municipal Councils of Montalegre and Boticas, as well as ADRAT (Associação de Desenvolvimento da Região do Alto Tâmega).

### Study procedure

**Fig. 1:**
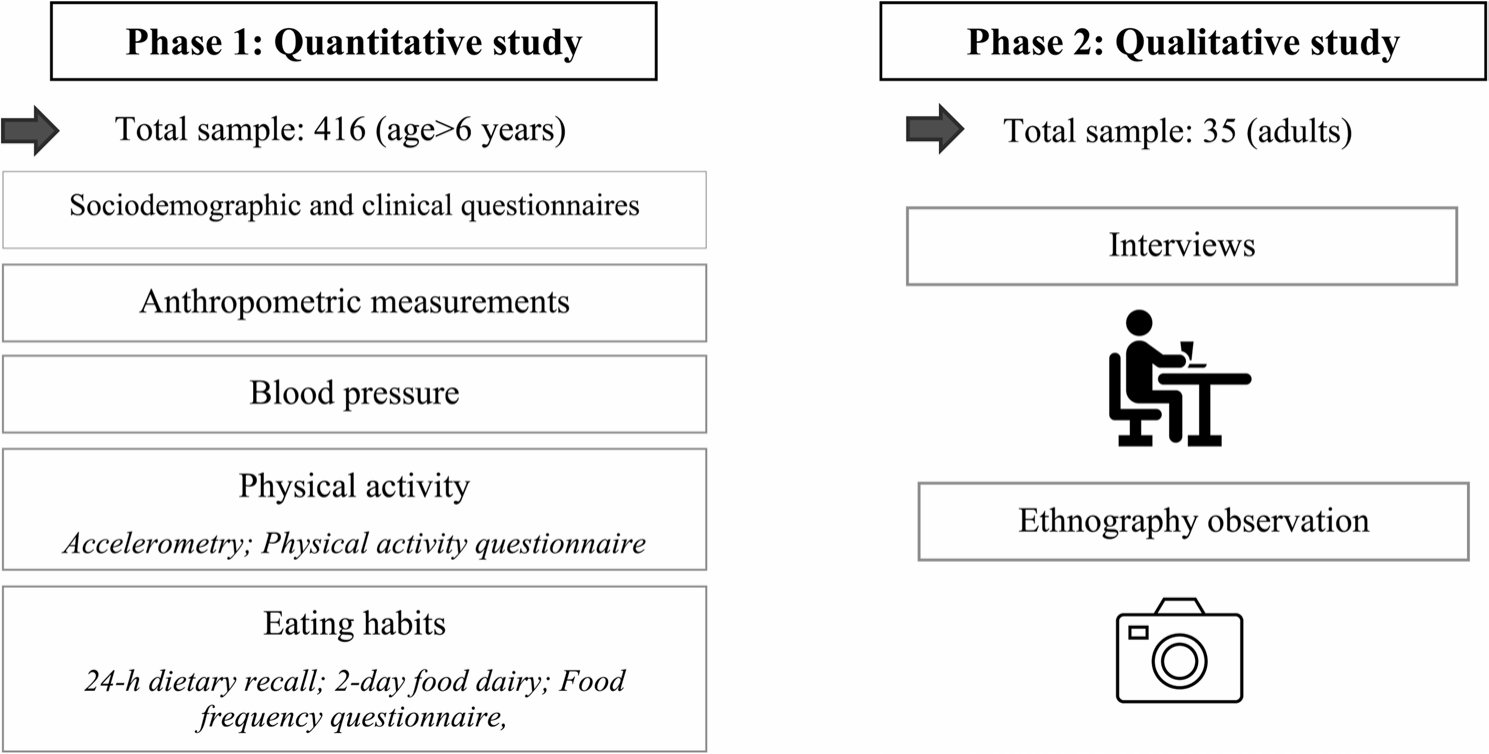
Overview of the mixed-method study design, including the planned quantitative and qualitative components, of the Valor Barroso study (Barroso, Portugal, 2025–2026)

As the project consists of mixed methods, the procedure descriptions are divided into quantitative and qualitative parts (Fig. 1).

#### Quantitative part

Phase 1 is a cross-sectional study involving 416 participants of different ages, from April 2025 to April 2026. The study will be conducted based on the Strengthening the Reporting of Observational Studies in Epidemiology (STROBE) Statement, a guideline for observational studies [20].

Assessments will be carried out in each civil parish of the municipalities of Montalegre and Boticas on dates previously scheduled that will be widely publicized to the local population. Data collection will place in predefined community locations, including municipal headquarters and other community spaces, such as civil parish council or schools, ensuring at least one visit to each civil parish.

The project will be promoted through local communication channels and institutions in partnership with the leaders of each community. This strategy aims to reduce barriers to access and promote greater public participation in the study.

Those interested who meet the eligibility criteria will be assessed. During the assessment, the study procedures will be explained, followed by the signing of informed consent. Participants will undergo a series of questionnaires and anthropometric assessments, which will take approximately 40 min. Additionally, they will receive a package containing accelerometers, along with detailed instructions on how to use them, and the 2-day food diary. At the end of the 7 days, they were instructed to return to the devices.

As this is a community-based study, the research team aims to engage entire families to ensure a comprehensive analysis, where possible, to capture a holistic view of the population’s habits. In cases where a family is willing to participate, all eligible family members (adults, children, and adolescents) will be considered for inclusion in the study.

#### Qualitative part

Phase 2 is a qualitative study that employs an ethnography collection, composed of semi-structured interviews and ethnographic observations. The interviews will be conducted with a subsample of participants, from September 2025 to March 2026, and the ethnographic observations will be conducted throughout the entire year of 2026.

The interviews will follow guidelines from the Consolidated Criteria for Reporting Qualitative Research (COREQ) [21]. The semi-structured schedule consists of open-ended questions with an inductive thematic analytical approach [22, 23]. Each interview session lasted between 30 and 60 min and will be recorded using a digital voice recorder and later transcribed. Prior, all participants will be required to read the participant’s information sheet and sign the informed consent.

Ethnographic participant observation will include photographic and video documentation. We will immerse ourselves in participants’ lives, spending time with them, participating in local food practices and daily activity. Video and photographic materials will be used in the production of documentary and educational resources. Participants will be explicitly informed about how their data will be used, ensuring that informed consent is obtained for the use and potential public dissemination of their images and videos.

### Participants

#### Barroso region

Barroso is a region located in northern Portugal and is composed of the municipalities of Montalegre and Boticas. It has a population of 14,027 individuals, 52% are female and 48% are male [18]. The region faces significant demographic challenges, common to other rural areas in the country and Europe, with one of the highest ageing rates in Portugal: more than 40% of the population is aged 65 years or over, whereas the national average of 23%, with a ratio of 509 elderly people for every 100 young people [18].

#### Eligibility criteria

Inclusion criteria:

- Men, women, children, and adolescents.
- Age: >6 years.
- Native and permanent residents in the Barroso region.
- Written informed consent prior to participation by adults or by parents/legal caregivers for children/adolescents.
- Availability to participate in all proposed assessments.

Exclusion criteria:

- They are considered dependent on long-term care.
- Has any disability that may affect the basic activities of daily living.
- Having been diagnosed with dementia, developmental disorders or another mental illness that inhibits a complete understanding of the study.
- Acute or chronic severe health conditions that interfere with dietary or physical activity patterns.

#### Sample size

Using the estimated population size of 14,027 in Barroso [18], the representative sample of the population was calculated considering a confidence level of 95% and a statistical error of 5%, following the equation:

$$n=\frac{N*{Z}^{2}*p*(1-p)}{\left(N-1\right)*{d}^{2}+{Z}^{2}*p*(1-p)}$$

*n = sample size; N = total population (14027); Z = confidence level (95%=1.96); p = expected proportion (50%=0.5); d = margin of error (5%=0.05)*.

The resulting sample size was 374 participants. To consider possible dropouts, it was decided to increase these to 416 participants. The proportions of men and women and the distribution across age groups reflect the local population structure, based on official demographic data from the National Institute of Statistics for the Barroso region [18]. This approach aims to enhance the representativeness of the study sample. (Table 1).

**Table 1:** Target sample stratification by age group and sex showing the planned number of participants enrolled in the quantitative phase of the Valor Barroso study (Barroso, Portugal)

Age group (years)	Total in sample	Men	Women
0–9	22	11	11
10–19	26	13	13
20–39	64	32	32
40–64	138	70	68
> 65	166	75	91

For qualitative analysis, a subsample selected from the quantitative sample will be selected through informal contact and snowball sampling to ensure the inclusion of individuals with diverse demographics and backgrounds. Data collection will continue until thematic saturation is reached, with an estimated 25–35 interviews expected [24].

### Data collection

#### Quantitative data

The data will be collected by the main investigator and the research team, which will include qualified nutritionists and exercise physiologists, follow a standardized protocol and aim to ensure consistency and reliability during the study. For children under 12 years of age, parents or caregivers are responsible for completing both documents together with the child. Older children (12 to 18 years of age) self-reported their eating habits and most of the individual variables (clinical, sociodemographic, and questionnaires) but may also receive assistance from their parents or caregivers [5, 25].

##### Sociodemographic and clinical variables

The participants will be asked about sociodemographic variables, including age: gender (male, female, or not answer/other); nationality; marital status; educational level (elementary or less, secondary, university or higher education); and professional status, occupation and work characteristics (specifically for participants working in agriculture or farms) [26].

A personal and family history of diagnosed diseases will be collected (including diabetes, dyslipidemia, arterial hypertension, heart diseases, oncological diseases, thyroid illnesses, hyperuricemia, osteoporosis, bone fractures, chronic obstructive pulmonary disease, ulcers/gastritis, gallstones, diverticular disease, hepatitis, and nephropathy) [27]. Additionally, participants will be requested to provide a record of the medications and supplements (prescription and over the counter) they are currently taking, including doses and amounts. Smokers are queried about their smoking habits (frequency and quantity) encompassing cigarettes, electronic cigarettes, cigars, pipes, smokeless tobacco, and/or snuffs, and participants will also be asked about the frequency and quantity of their standard alcoholic beverage intake [28].

The health-related quality of life of participants will be assessed using two instruments validated for Portuguese and tailored to different age groups: the 36-Item Short Form Health Survey (SF-36) [29], which is administered to adults aged 18 years and older, and the KIDSCREEN-10 Index, developed specifically for children and adolescents [30]. Furthermore, the Multidimensional Scale of Perceived Social Support (MSPSS) [31] will be used to assess subjective social support. This Likert-type scale consists of 12 items across three domains: family, friends, and significant others, and provides insights into the perceived social support levels of participants. Sleep quality was assessed with the Snyder scale, a single-item Likert scale [32].

##### Anthropometric

Anthropometric assessment will be conducted following the protocols and quality standards established by the International Society for Advancement of Kinanthropometry (ISAK), which will be performed by trained nutritionists [33]. All measurements will be obtained using appropriate age procedures, settings, and reference standards for children, adolescents, adults, and older adults, to ensure valid assessments across the different age groups [33].

The following anthropometric measurements will be collected: patient height (averaging two measurements) using a Seca-213 floor-standing stadiometer; waist circumference, which is determined by locating the midpoint between the upper edge of the iliac bone and the lower edge of the ribs; and hip circumference, which is measured at the largest circumference around the buttocks, via a nonelastic measuring tape with one decimal place [34]. Body composition assessment (fat body mass, truncal fat mass, abdominal fat mass, and lean body mass) will be performed using the InBody Model 770 bioimpedance scale.

Weight and height will be measured by a health professional using standard procedures and equipment as part of the health examination. Body mass index (BMI) will be calculated as weight/height^2^ (kg/m^2^). For children and adolescents, BMI will be interpreted using age and sex specific percentiles based on WHO growth reference standards [34]. For adults and older adults, BMI classification will follow standard adult cut-off values recommended by the WHO [34].

##### Blood pressure

Blood pressure will be measured by a qualified professional with a digital blood pressure monitor following a standardized and age appropriate protocol [35]. Measurements will be performed using cuffs of appropriate size according to participants’ arm circumference and age.

The participants will sit with their legs uncrossed and their backs supported and will be instructed to relax and not talk during a 5-minute rest period or during the measurement. The process will be repeated at least twice, and the average of the two readings will be recorded for analysis, with classification according to standard guidelines [35]. If either systolic or diastolic measurements differ by more than 5 mmHg between measurements, a third measurement will be taken.

Blood pressure values will be interpreted according to age specific reference standards. For children and adolescents, classification will be based on sex, age, and height percentiles. For adults and older adults, blood pressure will be classified according to standard clinical guidelines.

##### Physical activity

##### Accelerometry

To assess physical activity and sedentary behavior, participants will wear the triaxial accelerometer wGT3X+ (Actigraph, USA) [36], for seven consecutive days. To ensure correct use of the device, participants will receive detailed information, both orally and in writing, on how to properly use the accelerometer. The participants will be instructed to wear the accelerometer on a non-dominant wrist for seven consecutive days, throughout all waking hours, and will be advised to remove it during bathing, or water-related activities.

The accelerometer will be calibrated and synchronized to record triaxial accelerations at 55 Hz [36], and epoch length of 60 s [37]. The study will include only individuals who have a minimum usage of the device for at least 3 days during the week and 1 day during the weekend, with a minimum of > 10 h of daily usage [37–39]. Accelerometer wear time will be determined based on activity counts per minute. Non-wear time is defined as 90 min or more of consecutive zero activity counts, with a spike tolerance of 2 min and 100 counts per minute [40]. Data from the accelerometer will be processed in ActiLife Data Analysis Software (V6.13, Pensacola, USA).

##### Physical activity questionnaire

The long-version International Physical Activity Questionnaire (IPAQ) [41] will be applied to adults to assess the participants’ usual level of physical activity. It records physical activity at four intensity levels: vigorous, moderate, walking, and sitting. For children aged 6 to 12 years, and adolescents aged 12 to 18 years, the Physical Activity Questionnaire for Children (PAQ-C) and the Physical Activity Questionnaire for Adolescents (PAQ-A) [42] will be used, respectively. These adapted questionnaires are specifically designed to assess the physical activity patterns of younger individuals and provide information on activity levels across different intensities, including sedentary behaviors.

##### Eating habits

Eating habits will be assessed using a combination of complementary methods: Food Frequency Questionnaire (FFQ), 24-hour dietary recalls, and a 2-day food dairy. The use of multiple dietary assessment tools allows capturing different dimensions of eating behavior, improves the accuracy of dietary intake estimation, and minimizes the limitations inherent to any single method. This combination allows capturing short-term intake, daily variability, and defining habitual dietary patterns.

##### Food frequency questionnaire (FFQ)

A trained nutritionist will administer the FFQ, which has 89 items and has been validated for the Portuguese population [43]. The FFQ is a quantitative method used to assess participants’ usual dietary patterns over the past 12 months. The participants will answered questions about the frequency of consumption of different food groups, including foods and beverages commonly consumed in Portugal.

##### 24-h dietary recall

The 24-h dietary recall will be conducted by a previously trained and standardized nutritionist. Participants provided detailed information about all the foods and beverages consumed on the day prior to the interview, including portion sizes, cooking methods, and time and location of consumption [44]. The recorded data will be used to estimate total food consumption, via the Food Composition Table - Instituto Nacional de Saúde Doutor Ricardo Jorge [45].

##### Food diary

For the food diary, participants will complete a 2-day food record (one weekday and one weekend day). The participants will be instructed on how to carefully fill out the record that they will receive printed. The form will include fields for participants to note and estimate the details of all meals, snacks and beverages consumed, including the time of consumption, household portion sizes and cooking methods. For meals eaten in restaurants, participants will be asked to record the name of the restaurant and the dishes and beverages consumed, including water, tea, coffee, sugary and alcoholic drinks. The recorded data will be analyzed by trained dietitians, through a food diary guide specially designed for this study, and used to estimate total energy and nutrient intake, via the Food Composition Table - Instituto Nacional de Saúde Doutor Ricardo Jorge [45].

#### Qualitative data

This study uses a focused ethnographic approach for our exploration. The main researcher led the interviews to ensure that the data collection and interpretation were consistent. The interview will be following a predefined semi-structured schedule with open-ended questions, based on literature reviews [46–50]. The schedule ensures that all questions are covered; however, we also encourage participants to share their stories, offering rich insights and experiences.

The questions cover the following topics: (1) traditional and cultural foods, along with food practices, and (2) physical activity routines and habits, as shown in Table 2.

**Table 2:** Thematic domains and guiding questions for semi-structured interviews conducted in the qualitative phase of the Valos Barroso study (Barroso, Portugal)

Traditional and cultural foods	***Commonly consumed foods*** “What foods do you eat every day?” “What is a typical meal in your house?” ***Traditional dishes and preparation methods*** “What traditional dishes do you usually make? How do you prepare them?” “Is there any food that is unique to Barroso?” ***Rituals and social aspects of food consumption*** “Who usually cooks in the community?” “What dates are celebrated in your village/family?” “What is typically served during festivals or celebrations?” ***Changes over time*** “Have you noticed changes in your eating habits over the years? What were meals like in the past?” ***Economic or social Impacts*** “Has anything changed in the food you eat due to money or difficulty finding ingredients? Have there been times when you couldn’t buy certain foods?
Physical activity routines and habits	***Habits and routines*** *“What physical activity do you do on a daily basis?”* *“How much physical activity do you do for fun*,* leisure*,* or as part of your work?”* ***Traditional community activities*** *“In your community*,* are there dances*,* games*,* or events that help people stay active or have fun? What are they?”* ***Changes in work practices and physical activity over time*** *“What were the physical activities or work people were doing in the past? Has anything changed in how people work or move?”* *“Does agricultural or pastoral work fit into your routine? Are there times of the year when this work is intense?”* *“Do you think younger people are more active or exhibit different movement patterns than older people?”* ***Social and environmental impacts*** *“Does the terrain*,* climate*,* and distance affect how you move day to day?”* *“Do you usually walk in green areas*,* like parks or trails? How do these places help you?”*

For ethnography observation, we will immerse ourselves in the daily lives of participants. We will engage with their environment and join in local food practices and activities. This will help explore how behaviors, beliefs, values, and emotions shaped in specific sociocultural contexts [51]. These observations will include photographic and video documentation. We plan to participate in weekly markets and municipal festivals, where locals showcase their products; Roundtable with representatives of the community and the researcher speak about their lifestyle; Culinary processes in traditional kitchens; Comply the daily work routine of the farmers and agriculture. These materials will be used to create documentaries and educational resources. Field notes capture subtle details that interviews might miss, such as non-verbal cues and how the environment affects food and physical activity practices.

### Ethical aspects and consent

The research will be conducted in accordance with the Declaration of Helsinki and comply with national and EU legislation regarding medical research with human subjects. It will also meet the General Data Protection Regulation (Regulation (EU) 2016/679).

Participants will receive a detailed explanation of the study objectives, procedures, and possible risks and benefits. Their participation will be confirmed once both the participant, and the researcher sign the informed consent form. Each participant will receive a printed copy of their records.

For adults, participation in the study will only occur after the participant has signed the informed consent form. For children and adolescents under 18, consent will be obtained from their parents or caregivers, who will sign the consent form on behalf of the child. Additionally, children and adolescents will be asked to provide their own assent to participate, ensuring that they understand the study's purpose and procedures in an age-appropriate manner.

The participants chosen for qualitative analysis will need to provide additional written permission for the use of audio, video, and photos collected during the study, and the possible public dissemination of the edited videos for educational and research purposes.

### Data management and analysis

#### Quantitative data

We will collect quantitative data using both paper (food diaries) and electronic forms. Data will be digitized using a Microsoft Excel spreadsheet and stored on a secure server at the Instituto Politécnico de Bragança. We use cloud technology and follow data protection regulations. Each participant will receive a unique numeric code to ensure anonymity. Researchers will not access identifiable participant personal data.

We will process accelerometer data with ActiLife Data Analysis Software. This software classifies sedentary time and different levels of physical activity using validated cutoff points for various age groups [52,53].

Descriptive analysis will be conducted via SPSS software, and the means ± standard deviations (SDs) for continuous variables and absolute and relative frequencies (n and %) for categorical variables will be calculated. This analysis will be stratified by sex, age group, and other relevant sociodemographic characteristics. The patterns and associations among dietary habits, physical activity levels, and health markers were subsequently identified. To explore these associations, correlation analyses and regression models, adjusted for potential confounding variables will be used. Finally, stratified analyses will be conducted to examine how these factors vary across different age groups (children, adolescents, adults, and older adults) to account for possible age-related differences in dietary and physical activity patterns.

#### Qualitative data

Qualitative data from the interviews will be transcribed from the audio records and analyzed using NVivo software, which will allow for the organization, coding, and identification of patterns.

The initial coding will be carried out in an open way, identifying relevant excerpts for the research questions. The generated codes are grouped into themes and subthemes. We consider the theoretical saturation reached when no new code emerges from the data.

## Discussion

The main objective of this study was to characterize the lifestyle patterns, with a focus on dietary habits and physical activity, of the Barroso population. This region, consisting of the municipalities of Montalegre and Boticas, is predominantly rural and is known for its strong agricultural tradition and preservation of cultural practices, which include folk festivals, traditional cuisine, and physical activities related to fieldwork. These characteristics may directly influence the dietary behaviors and physical activity patterns of the local population but have been poorly studied thus far.

The physical activity pattern is multifaceted, involving not only recreational exercise but also fieldwork, domestic tasks, and active transport. The accurate assessment of these different components represents a methodological challenge, particularly in epidemiological studies that rely on self-reported instruments, which may be subject to memory and interpretation biases. In this context, the study will use accelerometers, equipment which are increasingly adopted as objective tools to quantify body movement, enable a rigorous analysis of physical activity patterns and their relationship with other health behaviors. With respect to nutrition, the study will assess dietary habits using a multi-method approach, including 24-hour dietary recall, food frequency questionnaires, and food diaries. This approach allows the assessment of habitual intake, short-term consumption, and daily variability, thereby strengthening the robustness and validity of dietary data.

The innovation of this study lies in the combination of these quantitative assessments with a qualitative analysis through an ethnographic approach that involves observations, photographs, videos, and interviews, enabling a deeper understanding of the cultural and social aspects that shape the dietary habits and lifestyle of the region. Ethnography plays a crucial role in this type of research as it provides a more detailed perspective on human behavior, allowing the exploration of the complex interactions among culture, society, and health.

A comprehensive scientific publication will be prepared, detailing the dietary habits and physical activity practices of the local population, providing information on regional food culture, traditions, physical practices associated with the environment, modern influences, and the challenges faced by the local community.

The results of this cross-sectional mixed-methods study are intended to inform and guide the development of culturally sensitive public health strategies. By identifying lifestyle patterns in the region, community needs, and potential areas for action, this research can support future initiatives aimed at promoting healthier lifestyles in the Barroso region, respecting and valuing its unique cultural and agricultural heritage.

However, the study has limitations. Given the community-based recruitment strategy, which relies on dissemination through on local communication channels and voluntary participation, the study sample may not fully represent the entire population of Barroso, despite stratification by sex and age. Therefore, the results should be interpreted considering potential selection bias. However, the study will provide valuable information on lifestyle patterns within a diverse segment of the local population and will help address an important knowledge gap in this under-researched region.

## Data Availability

Not applicable.

## Abbreviations

NCD: Non-communicable diseases
GIAHS: Globally Important Agricultural Heritage System
FAO: United Nations Food and Agriculture Organization
CIMO: Centro de Investigação da Montanha
SUSTEC: Laboratório Associado para a Sustentabilidade e Tecnologia em Regiões de Montanha
CIAFEL: Centro de Investigação em Actividade Física, Saúde e Lazer
ITR: Laboratório para a Investigação Integrativa e Translacional em Saúde Populacional
ICF: Informed consent form
IPAQ: International Physical Activity Questionnaire
MET: Metabolic Equivalent of Task
PAQ-C: Physical Activity Questionnaire for Children
SF-36: 36-Item Short Form Health Survey
CHQ-PF50: 50-items Child Health Questionnaire
BMI: Body mass index (BMI)
ISAK: International Society for Advancement of Kinanthropometry
WHO: World Health Organization
MSPSS: Multidimensional Scale of Perceived Social Support
FFQ: Food Frequency Questionnaire
SFAs: Saturated fatty acids
MUFAs: Monounsaturated fatty acids
PUFAs: Polyunsaturated fatty acids
SDs: Standard deviations
